# The Programming Optimization of Capacitorless 1T DRAM Based on the Dual-Gate TFET

**DOI:** 10.1186/s11671-017-2294-3

**Published:** 2017-09-06

**Authors:** Wei Li, Hongxia Liu, Shulong Wang, Shupeng Chen, Qianqiong Wang

**Affiliations:** Key Laboratory for Wide Band Gap Semiconductor Materials and Devices of Education, School of Microelectronics, Xidian University, Xi’an, 710071 China

**Keywords:** Tunneling FET (TFET), DRAM, Programming optimization, Retention time

## Abstract

The larger volume of capacitor and higher leakage current of transistor have become the inherent disadvantages for the traditional one transistor (1T)-one capacitor (1C) dynamic random access memory (DRAM). Recently, the tunneling FET (TFET) is applied in DRAM cell due to the low off-state current and high switching ratio. The dual-gate TFET (DG-TFET) DRAM cell with the capacitorless structure has the superior performance-higher retention time (RT) and weak temperature dependence. But the performance of TFET DRAM cell is sensitive to programming condition. In this paper, the guideline of programming optimization is discussed in detail by using simulation tool—Silvaco Atlas. Both the writing and reading operations of DG-TFET DRAM depend on the band-to-band tunneling (BTBT). During the writing operation, the holes coming from BTBT governed by Gate2 are stored in potential well under Gate2. A small negative voltage is applied at Gate2 to retain holes for a long time during holding “1”. The BTBT governed by Gate1 mainly influences the reading current. Using the optimized programming condition, the DG-TFET DRAM obtains the higher current ratio of reading “1” to reading “0” (10^7^) and RT of more than 2 s. The higher RT reduces the refresh rate and dynamic power consumption of DRAM.

## Background

The dynamic random access memory (DRAM) has become as an integral memory cell in the mobile and computing system [1–3]. With the shrink of device geometrics, the large volume of capacitor is an inherent disadvantage for the traditional one transistor (1T)-one capacitor (1C) DRAM cell, which limits its large-scale application. The capacitorless 1T DRAM cell based on the floating-gate transistor has shown the potential advantage compared with the conventional 1T-1C DRAM for the high density packaging of memory [4]. In the floating-gate transistor, the charges in the substrate region are stored in the floating-gate region by the Fowler-Nordheim tunneling. And the reading operation depends on the thermionic emission [5, 6], which is the same as the metal-oxide-semiconductor field-effect transistor (MOSFET). As a result, the reading current of the DRAM with the floating-gate transistor has a strong dependence on the temperature. Furthermore, the thermionic emission causes the subthreshold swing (SS) of transistor to be higher than 60 mV/dec, which makes the high leakage current and power consumption become the major challenges [7–9].

Recently, the tunneling field-effect transistor (TFET) has been regarded as a promising candidate for the future low-power electrical devices [10–12]. The main conduction mechanism of TFET is band-to-band tunneling (BTBT) instead of thermionic emission, which makes it obtain the several advantages such as the sub-60 mV/dec SS, low off-state leakage current, and weak temperature dependence [13, 14]. So far, large amounts of research work about TFET mainly focuses on the study on the device performance of single TFET and some simple circuits consist of the TFETs. However, the high *I*_on_/*I*_off_ of the TFET enables it to serve for the DRAM cell [15]. Especially, the low off-state leakage current can reduce the reading “0” current and the power consumption of DRAM cell. The researchers have designed a dual-gate TFET (DG-TFET) DRAM with the capacitorless structure [16]. In the DG-TFET DRAM, the charge storage during the writing operation is based on the BTBT between the channel and drain, which is mainly produced by Gate2. At the same time, the tunneling of electrons promotes the accumulation of holes in channel region under Gate2. Gate1 is mainly responsible for reading operation. The reading current of DGTFET DRAM mainly relies on the BTBT between the source region and channel region. There are some research groups which have demonstrated that reading current of DG-TFET DRAM has a weak dependence on temperature. And DG-TFET DRAM can obtain a retention time of higher than target (64 ms) [17]. But the current ratio of reading “1” to reading “0” and RT are not the optimum value due to the un-optimized programming condition.

The performance of TFET DRAM, especially the current ratio of reading “1” to reading “0”, has a great dependence on programming condition. Gate2 mainly influences the BTBT during the writing operation, which dominates the storage region of charges and potential well under Gate2. Gate1 governs the BTBT during the reading operation, which mainly influences the reading “1” current. The proper biases of both Gate1 and Gate2 can make DGTFET DRAM obtain the higher current ratio. There is a little literature studying the influence of programing condition on reading current. In this paper, a detailed programming optimization guideline is proposed, including writing, holding, and reading operations. By applying the optimized programming condition, the DG-TFET DRAM obtains the optimum performance—the reading current ratio of up to 10^7^ and the RT of more than 2 s. And applying the optimized programming voltage, the reading “0” current is much lower than that reported in reference [16, 18], which is helpful for the reduction of the power consumption.

## Methods

**Fig. 1:**
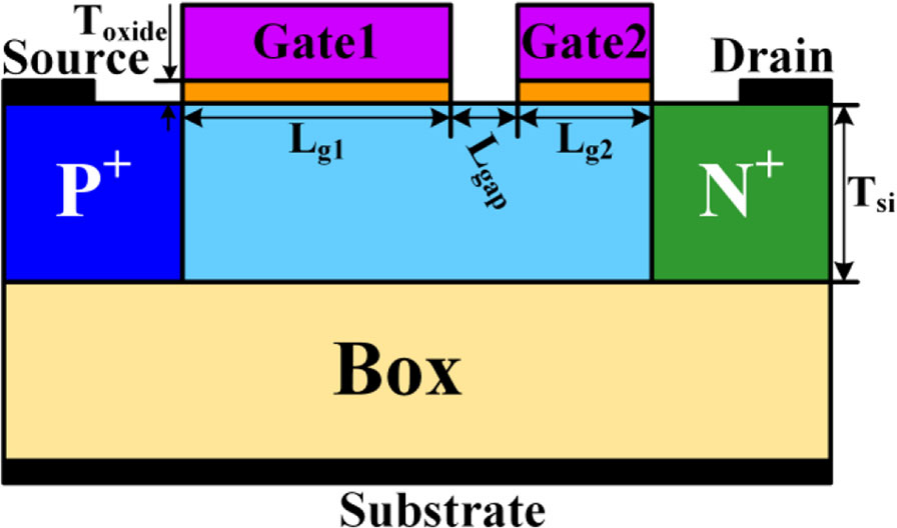
Schematic of DG-TFET DRAM cell. This figure shows the schematic of dual-gate TFET (DGTFET) DRAM cell, including Gate1, Gate2, source, drain, and channel. In this design, the source region and drain region are P^+^ doping and N^+^ doping, respectively. Gate1 and Gate2 are N^+^ polysilicon and P^+^ polysilicon, respectively

The structure of DG-TFET investigated in this paper is illustrated in Fig. 1. The doping concentration of both the P^+^ source and N^+^ drain is 1 × 10^20^/cm^3^. The intrinsic channel is divided into two segments: Gate1 and Gate 2, and there is a short gap between Gate1 and Gate2. Gate1 and Gate2 are N^+^ polysilicon and P^+^ polysilicon, respectively. The P^+^ polysilicon Gate2 can create as well as maintain the physical well for charge storage and to replace the conventional TFET-based DRAM that utilizes a P^+^ pocket region as the storage area. While for an N^+^ polysilicon Gate1, the hole concentration in underlap region between Gate1 and Gate2 is low, which is helpful for the reading operation. Thus, a P^+^ polysilicon Gate2 is opted to have a deeper storage region that could facilitate longer retention, while an N^+^ polysilicon Gate1 is selected to control the tunneling mechanism during reading operation [18].

The detailed device parameters in the simulations are as follows: the thickness of bulk silicon (*T*_si_) is 20 nm; the lengths of Gate1 (Lg_1_) and Gate2 (Lg_2_) are 400 nm and 200 nm, respectively; the length of gap (*L*_gap_) between Gate1 and Gate2 is 50 nm; the thickness of gate oxide (HfO_2_) (*T*_oxide_) is 3 nm. The optimized programming conditions are shown in Table 1. The optimization guidelines of programming conditions will be discussed in detail in the following sections.

**Table 1:** Optimized Programming Condition

Operation	*V*_g1_	*V*_g2_	*V*_d_	*V*_s_
Writing “1”	0 V	−1.3 V	0 V	0 V
Writing “0”	0 V	1.3 V	0 V	0 V
Holding	0 V	−0.2 V	0 V	0 V
Reading	1 V	0.8 V	1 V	0 V

All the analysis is carried out in Silvaco-Atlas tool using the Nonlocal BTBT model [19]. The Nonlocal BTBT takes into account the nonlocal generation of electrons and holes, so it can model tunneling process more accurately. The tunneling model parameters are calibrated according to the experimental data in the reference [20]. Moreover, physical models including Shockley-Read-Hall recombination, Fermi statistics as well as doping and electric field-dependent mobility are also used. According to the approaches of [16, 18], the electron and hole lifetimes are set to 100 ns. The default temperature is 300 K.

## Results and Discussion

The operating principle of the DG-TFET DRAM cell is different from that of the traditional DRAM. Both the writing and reading operations are based on the BTBT, but each of them has the different function. The BTBT during the writing “1” leads the holes to be stored in the potential well under Gate2, which can elevate the reading “1” current. During the reading operation, the drain current mainly depends on the BTBT near the source side. Furthermore, the two gates also act as the different roles: Gate1 and Gate2 mainly determine the reading operation and writing operation, respectively.

### Writing Operation

During the writing “1”, Gate2 with the negative bias will boost the energy band of channel under Gate2, which diminishes the barrier width and produces the BTBT between the channel and drain. And this negative Gate2 bias also induces a deep potential well under Gate2. Due to the tunneling of electrons from the channel to drain, the channel region under Gate2 is fully depleted and a lot of holes are accumulated in this potential well. During the writing “0”, Gate2 with the positive bias makes the holes expel from the potential well which recombines at the drain side [21].

**Fig. 2:**
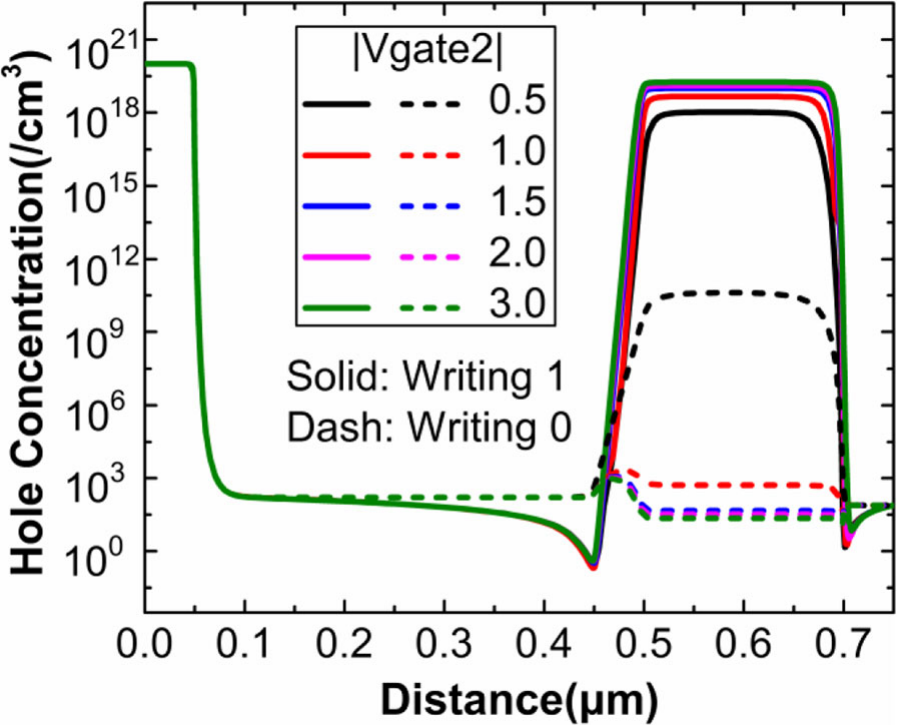
Hole concentrations on the surface of the channel after writing operation. This figure shows the variations of hole concentration with the different Gate2 voltages. The cutline is taken at the surface of the device from the source region to the drain region. In this figure, the solid line and dash line represent the hole concentration after writing “1” and writing “0”, respectively

Generally, the absolute value of Gate2 voltage keeps unchanged for the writing “1” and writing “0”. Figure 2 shows the variation of hole concentration with the Gate2 voltage after the writing operation. When the Gate2 voltage is 0.5 V, the hole concentration after writing “0” is higher due to the presence of potential well, which is detrimental for the state “0”. When the absolute value of the Gate2 voltage is higher than 1 V, the hole concentration after both writing “0” and writing “1” has no obvious variation. It suggests that BTBT is saturated for writing “1” and that all the accumulated holes during writing “1” expel from the potential well after writing “0”. And the difference of hole concentration between writing “1” and writing “0” is very evident, which is beneficial to distinguishing between the state “1” and state “0”.

**Fig. 3:**
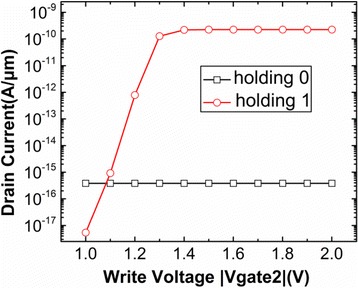
Drain current of DG-TFET after holding “0” and holding “1”. This figure shows the variation of drain current after holding “0” and holding “1” with respect to writing voltage

But the Gate2 bias during the writing operation cannot be only determined by the hole concentration. Figure 3 indicates that Gate2 bias during writing operation has the significant effect on the drain current after holding operation. The set of programming voltage during the holding operation will be discussed in the next section. Figure 3 reveals that the drain current after holding operation has no obvious variation when the absolute value of the writing voltage is higher than 1.3 V. Therefore, the −1.3 and 1.3 V are regarded as the optimal Gate2 voltage during the writing “1” and writing “0”, respectively.

**Fig. 4:**
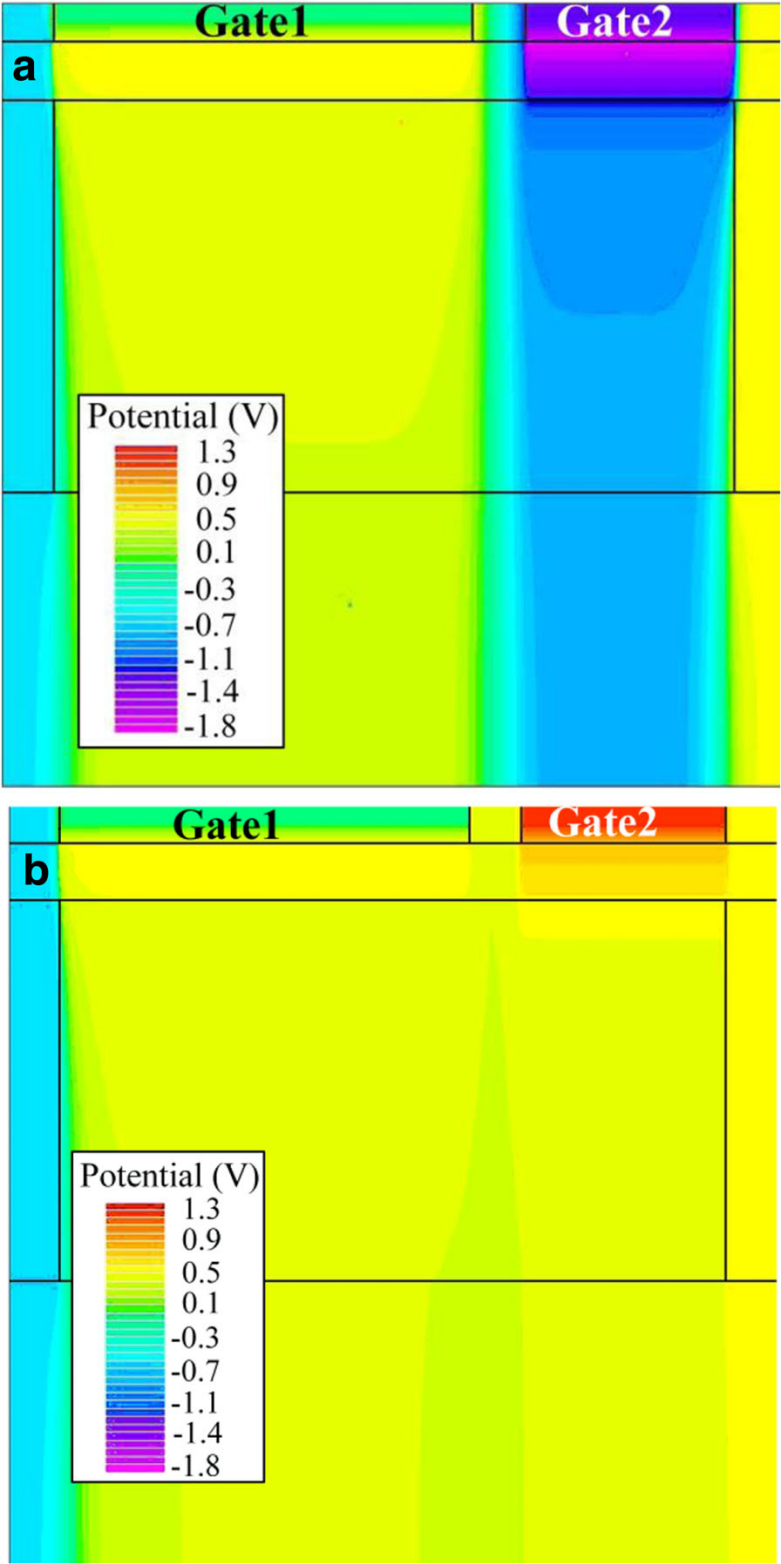
Potential contours after **a** writing “1” and **b** writing “0” when the absolute value of Gate2 voltage is 1.3 V. **a**, **b** The potential contours after writing “1” and writing “0”, respectively. The potential contours in this figure are extracted when the absolute value of Gate2 voltage is set to 1.3 V

Figure 4a, b respectively indicates the potential contour after the writing “1” and writing “0” when the absolute value of the writing voltage is 1.3 V. Obviously, a very deep potential well is created in the channel region under Gate2 after writing “1”, as shown in Fig. 4 a. The accumulated holes are preserved into this potential well during the writing “1”. However, the accumulated holes escape from this potential well during the writing “0”.

### Holding Operation

The holding process is mainly used to modify the retention of the charges. Usually, the zero bias is used during the holding operation in order to reduce the power consumption [22]. During the holding operation, the accumulated holes in potential well are gradually recombined due to the decreasing of the potential well depth. Therefore, the main purpose of optimization of holding operation is to avoid recombination of holes during holding “1”. In this design, a small negative bias is applied at Gate2 to retain holes in potential well after holding “1”, whereas the potential well is depleted of holes after holding “0”.

**Fig. 5:**
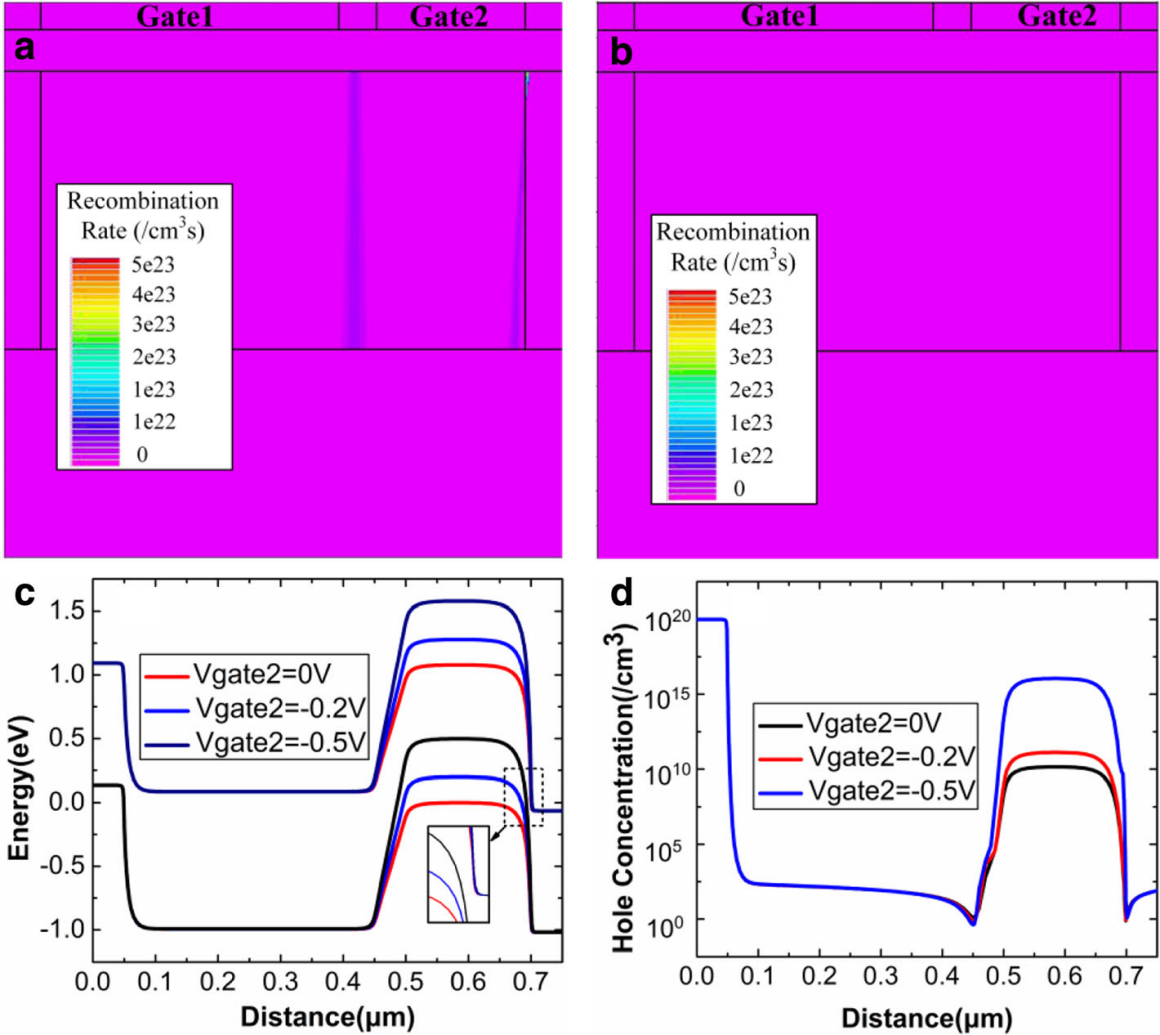
Recombination rate in DG-TFET DRAM cell after holding “1” when the Gate2 voltage is **a** 0 V and **b** −0.5 V; **c** energy band diagram and **d** hole concentration after holding “0”. **a**, **b** The recombination rate after holding “1” when the Gate2 voltage is set to 0 V and −0.5 V, respectively. **c** The energy band of device from the source region to drain region. **d** The hole concentration of the device after holing “0”. The energy band and hole concentration is extracted at the 3 nm below the gate oxide

With the more negative Gate2 voltage (−0.5 V) during the holding operation, the recombination of holes is eliminated after holding “1”, as shown in Fig. 5a, b. The elimination of hole recombination is beneficial for the retaining of holes during holding “1”. A small negative bias is applied at Gate2 to enhance potential well depth and retain holes for a long time, which is beneficial for the retention time of DGTFET DRAM. During holding “0”, a negative Gate2 bias can pull up the energy band of channel region under Gate2, which can prevent electrons coming from BTBT between the source and channel flowing towards drain side. Therefore, Gate2 with a negative can reduce the reading “0” current. However, Gate2 with more negative voltage (−0.5 V) diminishes the tunneling distance near drain side, as shown in Fig. 5c. This decreased tunneling distance causes the BTBT near the drain side during the holding “0”, which promotes the accumulation of holes in the potential well during the holding “0”, as shown in Fig. 5d. So the more negative Gate2 voltage (−0.5 V) during the holding “0” will degrade the state “0”. Therefore, in order to eliminate the hole recombination and BTBT during holding “1” and holding “0”, respectively, −0.2 V is regarded as the optimal Gate2 bias during the holding operation.

### Reading Operation

Subsequently, the optimization of reading operation is also investigated. The reading operation strongly relies on the BTBT between the P^+^ source and channel. During the reading “1”, Gate1 mainly promotes the BTBT at the source side, whereas Gate2 with the high voltage lowers the energy barrier which resists the flowing of electrons from the channel to drain. But during the reading “0”, it is necessary that Gate2 with the small voltage be able to prevent electrons flowing from the channel to drain. Therefore, the optimization of both the Gate1 and Gate2 voltages is very important for the reading operation.

**Fig. 6:**
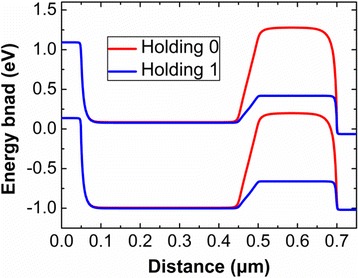
Energy band diagram after **a** holding “1” and **b** holding “0”. **a**, **b** The energy band of the device after holding “1” and holding “0”, respectively. The energy band is extracted at the 3 nm below the gate oxide

Figure 6 shows the different energy band diagrams after holding “1” and holding “0”. The same voltages are applied at the Gate sides during reading “1” and reading “0”. Since the positive Gate biases will be used during reading operation, the energy band will be put down whenever reading “1” or “0”. The energy band of channel under Gate2 after holding “0” is higher than that after holding “1”, and this energy band is also higher during reading “0” compared with that during reading “1”. The higher energy of channel under Gate2 will create an effective barrier to resist electrons flowing towards the drain side, which will decrease the reading “0” current.

**Fig. 7:**
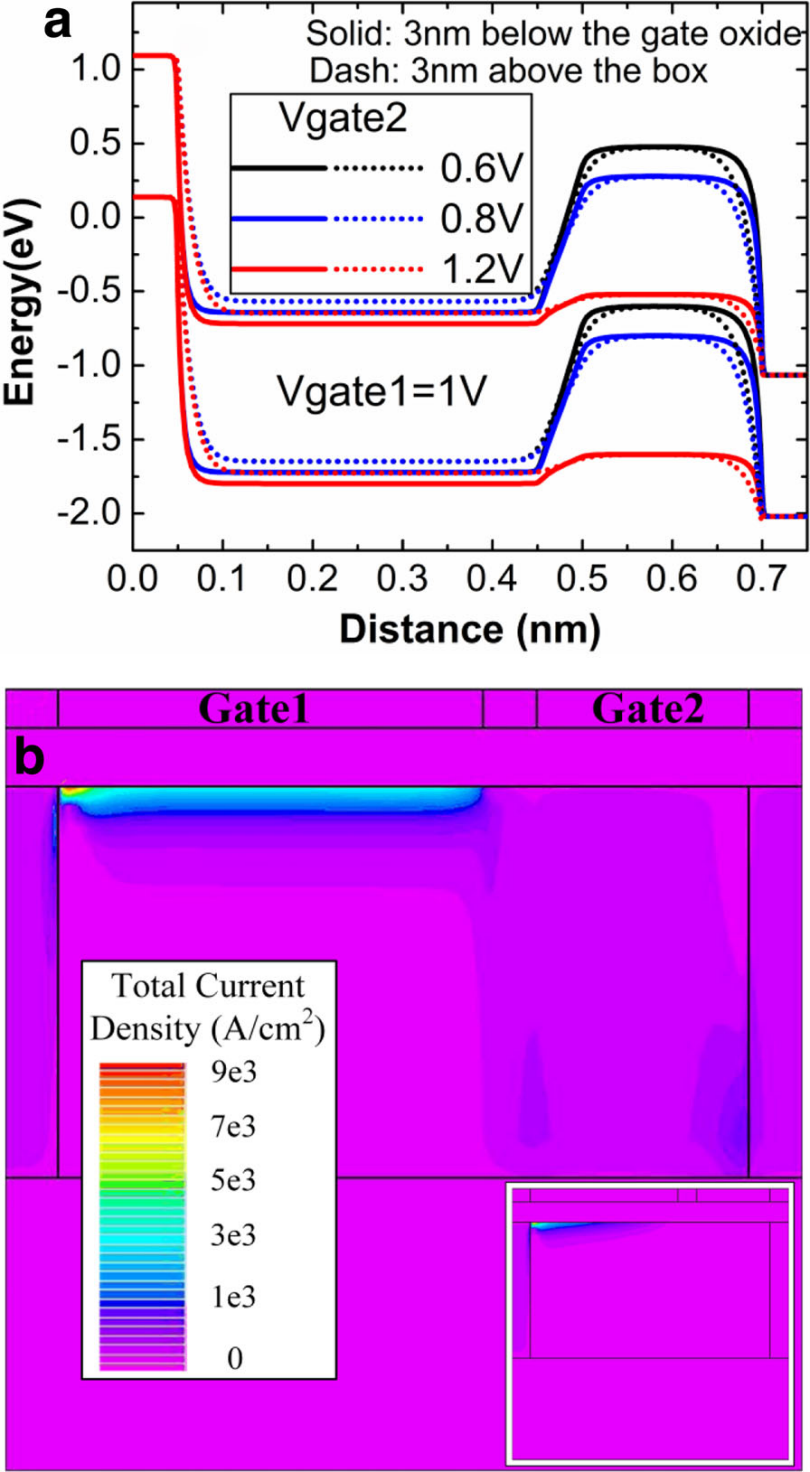
**a** Energy band diagram and **b** total current density after reading “0”. **a**, **b** The energy band and total current density after reading “0”, respectively. The energy band is extracted at the 3 nm below the gate oxide

During the reading operation, the drain bias is set to 1 V so that the drain current can be read in the DG-TFET. Firstly, keeping the Gate1 voltage of 1 V, change the bias of Gate2 to choose the optimal Gate2 voltage. Because the Gate2 voltage mainly influences the reading “0” current, the optimization of the Gate2 voltage is analyzed by the reading “0” mechanism. Figure 7a plots the variation of energy band with the Gate2 voltage after reading “0”. When the Gate2 voltage is lower (0.6 or 0.8 V), the channel under Gate2 becomes fully depleted. But when the Gate2 voltage rises to 1.2 V, the pull-down energy band of channel under Gate2 cannot create an effective barrier to prevent electrons flowing towards drain side. Figure 7b and its inset respectively show the total current density after reading “0” when the Gate2 voltage is 1V and 0.8 V. The obvious current density can be clearly found in the channel region under Gate2 when the Gate2 voltage is 1 V, which will give rise to the higher reading “0” current. So the 0.8 V is regarded as the optimal Gate2 voltage for the reading process.

**Fig. 8:**
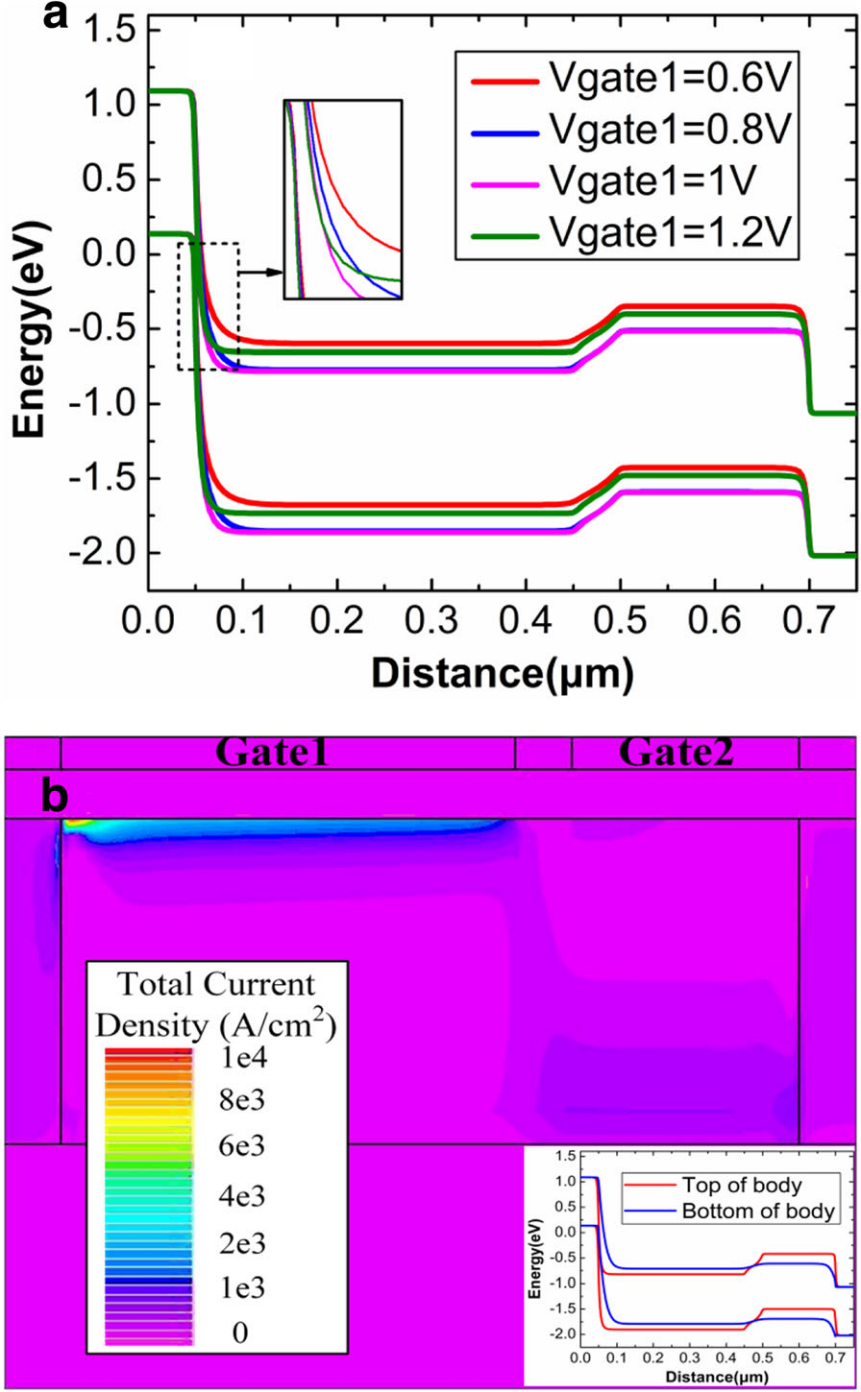
**a** Energy band diagram and **b** total current density after reading “1”. **a**, **b** The energy band and total current density after reading “1”, respectively. The energy band is extracted at the 3 nm below the gate oxide

Finally, the optimization of the Gate1 bias is also conducted. Figure 8a shows the variation of top energy band with the Gate1 voltage. The BTBT barrier width at the source side gradually decreases with the increasing of the Gate1 voltage, but this decreasing trend starts to saturate when the Gate1 voltage is higher than 1 V. And the Gate1 voltage of 1 V cannot bring sever influence on reading “0” operation, which has been demonstrated by Fig. 7b. Therefore, 1 V is regarded as the optimal Gate1 bias during the reading operation.

Through the above analysis, the deep potential well is only formed at the top of channel under Gate2 after writing “1”. Therefore, in the channel region under Gate2, the energy band at the top of channel is much higher than that at the bottom of channel. This demonstrates that there will be a barrier at the top of channel under Gate2 during the reading “1”. The inset of Fig. 8b plots the energy band at both the top and bottom of the channel after reading “1”. It can be clearly found that a higher channel barrier exists between Gate1 and Gate2 at the top of channel, but this barrier does not exist at the bottom of channel. Therefore, the conduction path is at the top of channel under Gate1 and the bottom of channel under Gate2 during the reading “1”, which can be clearly demonstrated by the current density in Fig. 8.

**Fig. 9:**
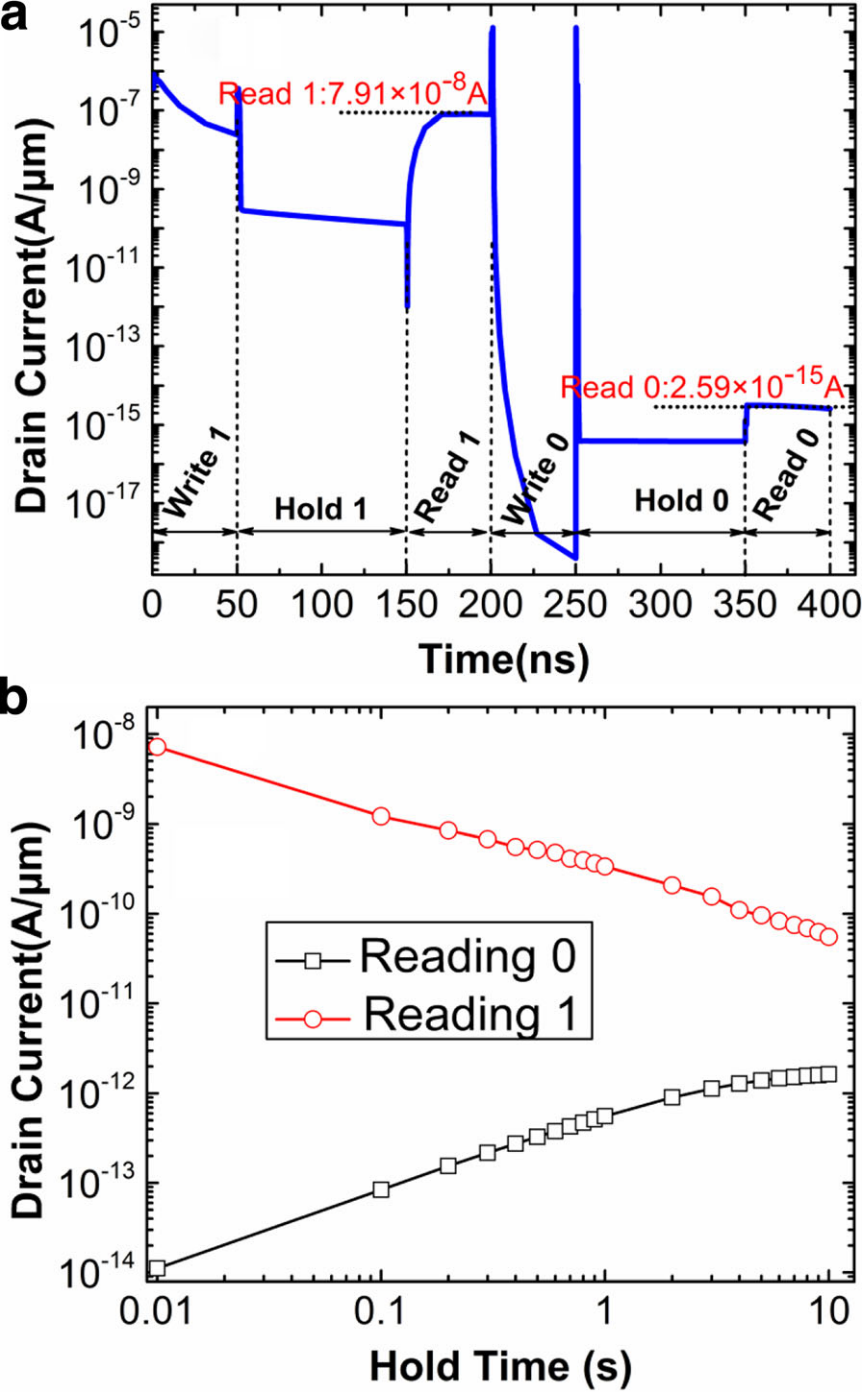
**a** Transient drain currents in the sequence of the operation; **b** variation of reading current with the holding time. **a** The transient current of DGTFET DRAM cell during the writing, holding and reading operations. **b** The variations of reading “1” and reading “0” current with the different holing times

Applying the above optimized programming condition, the transient response of DG-TFET DRAM cell is shown in Fig. 9. Both the writing and reading times are set to 50 ns, and the holding time is set to 100 ns. In Fig. 9a, the current ratio of reading “1” to reading “0” is as high as 10^7^, which is much higher than 10^2^~10^3^ in reference [16, 18, 23]. Furthermore, when the holding time rises to 10 s, the current ratio still exceeds 10. In reference [16], when the holding time is increased to 2 s, the current ratio is only about 10. Therefore, the RT of DG-TFET DRAM with the optimized programming condition is higher than 2 s. So, the optimized programming condition makes DG-TFET DRAM cell obtain not only the higher reading current ratio but also the larger RT. What is more, the reading “0” current with optimized programming voltage is much less than that in reference [16, 18, 22, 23], which enables it to meet the lower power application.

## Conclusions

The detailed optimization guideline of programming condition for the DG-TFET DRAM is proposed in this paper using the Silvaco-Atlas simulation tool. During the writing “1”, Gate2 with the negative voltage (−1.3 V) creates a potential well, and the BTBT between the channel and drain makes the holes be accumulated in this potential well. During the writing “0”, Gate2 with the positive voltage (1.3 V) makes holes escape form the potential well. For the holding operation, the small negative voltage (−0.2 V) is applied at Gate2 to retain the holes, which can improve the reading “1” current. After holding “0”, the barrier of channel under Gate2 can resist electrons flowing towards the drain side to reduce reading “0” current. For the optimization of reading operation, the larger Gate1 voltage (1 V) is mainly used to enhance BTBT at the source side during reading “1”, whereas an appropriate Gate2 voltage (0.8 V) is used to resist electrons flowing towards drain during reading “0”. The optimized programming condition makes the DG-TFET DRAM obtain the higher current ratio (10^7^) of reading “1” to reading “0” and retention time of more than 2 s. And the extremely low reading “0” current is helpful for the reduction of power consumption.
